# In the Long Run

**Published:** 1876-06

**Authors:** 


### IN THE LONG RUN

HONESTY is the best policy:
Temperance is the best life-preserver:
Rest is the best physic:
Carefulness is the best health protector:
Perseverance is the surest victor:
Kindness is the completest conqueror;
Difficulty is the best schoolmaster:
Experience is the best teacher:
Trouble is the best man-maker:
Frankness is the best friend:
Piety is the best practice.

On the other hand—roguery may for a while seem to prosper; self-indulgence may for a season be practiced with impunity; those who start ahead, as if they had not a moment to spare, may leave us to-day in the distance; the reckless may appear the gainers by their hardihood; and those who are quietly gliding down the stream of life on beds of roses, no obstacles opposing, no fears to quell, no tears to fall, no troubles to endure, no temptations to encounter, what are they? whence go they? and their work, what have they done? They never feed the hungry, nor clothe the naked, nor cheer the desponding, nor raise the fallen, nor nurse the sick, nor stand by the forsaken: no, no; their light goes out in early death; their memory rots during the time of their coevals; they made no mark in their short pilgrimage through the wilderness of time, to guide some after-coming brother in a safer or a better path; they did nothing for humanity, nothing for God; and their fate can be none other than to be by God and man forgotten. Stand up, then, my brothers, however poor, however low, however stricken; if want has ever wasted you, if oppression has ever ground you down, if slander, or fierce sickness, or crushing bereavements have almost wasted your life away; faith broken, confidence betrayed, motives impugned, and coldness, and ingratitude, and unreturned affections from nearest kindred, weigh heavily on the heart—what of it? It is the stroke of hardest steel that brings out the fire of life; it is the trampling on the flower which causes it to yield its richest perfume; it is the bursting of the bud that develops the full-blown rose in all its beauty; it is the fiery furnace that separates the purest gold from its unsightly dross. The rough and ponderous crowbar drives home into the hardest rock, and gives vent to living springs of purest water, when all else around is an arid desolation. It is the battle's contest that makes the conquering soldier; it is the strife for others life, through fire and flood, which brings out true heroism; and if in all this you have opposed fearlessness to danger, if you have met abuse with forbearance, if you have returned slander with silence, or gone nearer to the godlike, and apologized for the cursing SHIMEI, as did the man who was "after God's own heart;" if in crushing bereavements you have looked upwards through streaming eyes, and said, and felt, in loving resignation, "Thou did'st it;" or, if personal mortifications have been yours, and a PETER'S consciousness has come to you, and a PETER'S tears; if, when stricken down, even to prostration, you have arisen, and stood up, resolving to be still a man; if thus you have conducted yourself in all these, nor faltered, nor feared, nor fallen; have yielded to no impatience, have conferred not with temptation, have harbored no revenges under groundless defamings; have cherished no retaliations for malicious injuries; but have been steadily and lovingly trustful, that the sun would shine again and flowers bloom; and smile and song would lighten up the sky of the soul, and fill the heart with rapture, then have you cause for boasting, that although obscure, your fame has reached Heaven, that although friendless, you are the peer of angels.

